# Impaired *M*. *tuberculosis* Antigen-Specific IFN-γ Response without IL-17 Enhancement in Patients with Severe Cavitary Pulmonary Tuberculosis

**DOI:** 10.1371/journal.pone.0127087

**Published:** 2015-05-27

**Authors:** Lin Fan, Heping Xiao, Guangliang Mai, Bo Su, Joel Ernst, Zhongyi Hu

**Affiliations:** 1 Clinic and Research Center of Tuberculosis, Shanghai Key Lab of Tuberculosis, Shanghai Pulmonary Hospital, Tongji University School of Medicine, 200433, Shanghai, China; 2 Central Laboratory, Shanghai Pulmonary Hospital, Tongji University School of Medicine, 200433, Shanghai, China; 3 Division of Infectious Diseases, New York University School of Medicine, New York, New York, United States of America; Louisiana State University, UNITED STATES

## Abstract

**Background:**

Th1 cells play an essential role in immune protection against tuberculosis. Th17 cells might be involved with immune pathology in active human tuberculosis (TB). The balance between Th1 and Th17 cells in patients with cavitary tuberculosis needs to be clarified which might help understanding the immunological basis of pathologic pathogenesis in TB.

**Method:**

Initially treated pulmonary TB (PTB) patients with or without cavities were recruited before chemotherapy. We isolated peripheral blood mononuclear cells, stimulated with phytohemagglutinin (PHA), PPD, or ESAT-6 antigens, and assayed supernatant IFN-γ and IL-17 by ELISA after 24 or 72 hours incubation, respectively. Cells were also stained with antibodies to CD3, CD4, CD8, IFN-γ or IL-17 and the proportion of stained cells was measured by flow cytometry.

**Results:**

We found wide variation of IFN-γ response in active PTB patients, but less subject-to-subject variation of IL-17 was observed as we previously reported. There were no significant differences in IFN-γ and IL-17 between cavitary and non-cavitary PTB; however, we found decreased IFN-γ secretion in severe cavitary PTB compared to mild lesion non-cavitary PTB (p < 0.05). We also found a decrease in the proportion of CD3+CD4+ T cells in the blood of severe cavitary PTB patients (p < 0.05).

**Conclusions:**

IL-17 seemed to have no association with the formation of cavities in active PTB from the study of PBMC. Impaired IFN-γ without IL-17 enhancement occurs in peripheral blood during severe cavitary PTB. Our results demonstrate that *M*. *tuberculosis* antigen-specific Th1 response is decreased when PTB lesions develop to severe cavities.

## Introduction

Tuberculosis (TB) ranks as the second leading cause of death from the infectious diseases worldwide, there were 8.6 million new TB cases in 2012 as reported [1]. The bacterium can remain latent in the host, turn into active TB, or the pathogens can be eradicated through the interaction of the host’s immune system with *M*. *tuberculosis* [2]. Adaptive immune responses mediated by CD4+ T cells play a critical role in controlling the progress of *M*. *tuberculosis* infection, as HIV/AIDS patients whose CD4+ T cells are deficient are more susceptible to *M*. *tuberculosis*(MTB) infection than are HIV-uninfected individuals [3,4]. Moreover, the incidence of active tuberculosis in HIV-infected individuals is inversely proportional to the number of CD4 T cells in their peripheral blood [5, 6].

In addition to the roles of CD4+ T cells in immune protection against tuberculosis in humans, *M*. *tuberculosis* antigen-specific CD4+ T cells may contribute to immunopathology in the lungs and other organs [7,8]. Studies have demonstrated that in HIV-infected patients with active tuberculosis, the frequency of cavitary lung lesions is directly proportional to the number of peripheral blood CD4 T cells at the time of TB diagnosis [9]. One study revealed that a predominant Th1 immune response had been observed in non-cavitary tuberculosis patients while cavitary-involved segments of the lungs accumulated Th2 CD4+ T cell subsets [10]. Another study showed that neutrophils were predominant in the bronchoalveolar lavage fluid of cavitary lesions of tuberculosis [11], which is one of several potential pathogenic mechanisms including a dominant Th17 response [12].

Th17 cells, a relatively newly-described subset of CD4+ T cells, are implicated in host defense and in autoimmunity. IL-23 or IL-6, in combination with IL-1β, can efficiently induce IL-17 production in naive precursors, independently of TGF-β [13]. Recent studies showed that IL-17 contributes to both immune protection and immune pathology of tuberculosis [14, 15]. Some studies had showed that antigen specific IL-17 was involved with immune pathology in MTB infection. One study found that IL-17A is involved in the formation of mature granuloma in the Mycobacterium bovis bacille Calmette-Guérin (BCG)-infected lung [16]. Another study reported that MTB antigen-specific, IL-17-mediated neutrophil recruitment was directly related to increased pathological damage in the lung and enhanced pathological consequences are associated with an IL-17-dominated response and increased tissue involvement [17]. IFN-γ, mainly produced by CD4+ T cells, plays a critical role in resistance to tuberculosis and is necessary for host survival and optimal long-term protection against *M*. *tuberculosis* [18,19,20].

Our previous study showed that the variation of *M*. *tuberculosis* antigen-specific IFN-γ and IFN-γ /IL-17 exceeds that of responses to the polyclonal stimulus PHA in TST positive healthy humans, implying a variation of IFN-γ /IL-17 might be associated with outcome of infection in patients with active PTB [21]. Cavity is one of the common tuberculous lesions in lungs which can develop from caseating granulomas [22]. Therefore, we hypothesized that there exists an imbalance of Th1/Th17 in cavitary PTB: IL-17 might participate in the formation of pulmonary cavities in tuberculosis; immune protection might decrease while immune pathology increases in cavitary tuberculosis; and Th1/Th17 in cavitary PTB might be lower than in non-cavitary PTB. To determine our hypothesis, we quantified *M*. *tuberculosis* antigen-specific IFN-γ and IL-17 in peripheral blood and stained PBMCs with anti-human T cell antibody in patients with active PTB, looking for an association between cavity formation and Th1/Th17 response by T cells.

## Materials and Methods

### Study population

Subjects were recruited from patients hospitalized at Unit 1 in the Department of Tuberculosis, Shanghai Pulmonary Hospital between July 1, 2012 and December 31, 2013. Patients were included in the study if they fulfilled all the following criteria: ⑴diagnosed as newly treated active PTB confirmed by bacteriology, with all information regarding bacteriology, pathology, typical radiological manifestation and clinical response to anti-tuberculosis treatment available; ⑵ Tuberculous cavity or non-cavitary lesions in lungs could be observed on chest CT scan; ⑶ patients had never been treated for TB or treatment duration was within one week; and⑷ subjects provided informed consent for the study. During the same period, healthy volunteers were recruited for the control group. All procedures were performed in accord with the protocol, and this study was confirmed to be approved by the Ethics Committee of Shanghai Pulmonary Hospital (IRB number 2012017) which is the name of institutional review board in the hospital, written informed consent was obtained from all participants who signed and agreed to participate in this study.

All information regarding characteristics of thoracic radiology (diameter in cavity, number and location of cavities, symptoms, complications, history of BCG vaccination, disseminated extrapulmonary tuberculosis) was carefully recorded.

### Isolation of PBMC and cell culture assays

Venous blood samples (10 mL) were collected in heparin tubes; PBMC (peripheral blood mononuclear cells) were isolated within 3 hours after blood collection. PBMC isolation was performed by density gradient centrifugation using Ficoll-Hypaque (Axis-shield) following the manufacturer’s instructions. After washing the cells, 3x10^5^ cells per well were cultured in 200 μL medium of RPMI-1640 (GIBCO, Invitrogen) with 10% human serum, penicillin (50 U/mL), streptomycin (50 μg/mL), 2 mM L-glutamine and 10 mM HEPES using 96-well round-bottom plates (Costar). PBMC were then stimulated with PHA (phytohemagglutinin, a nonspecific T cell stimulus;5 ug/ml), PPD (purified protein derivative, 5 μg/mL, Statens Serum Institute, Denmark), recombinant ESAT-6 (early secreted antigenic target, 6 kDa of *M*. *tuberculosis*; 10 μg/mL), or medium alone as a negative control. ESAT-6 was recombinant and kindly provided by the Shanghai Institute of Immunology, Shanghai JiaoTong University School of Medicine.

### Cell staining and flow cytometry

PBMC were incubated with PPD, ESAT-6, PHA or medium alone at 37°C in 5% CO_2_ for 72 hours. Brefeldin A (eBioscience, BFA, final working concentration 3uL/mL) was added to the cells 4 hours before staining. Antibodies were prepared, cells transferred to tubes then spun down at 750 g for 5 minutes at 4°C and washed once in 500 uL FACS buffer. Samples were resuspended in 100 uL of diluted antibody against human cell-surface markers (CD4-PerCP-Cy5.5 (eBioscience,USA), CD8-PE/CYTM7 (BD,USA), or CD3-ECD (Beckman Coulter, France)), incubated for 30 minutes at 4°C, and washed twice in 500 uL FACS buffer. Supernatants were removed and cells were resuspended in 100 uL of Cytofix/Cytoperm(BD,USA), then incubated 20 minutes at 4°C. After washing cells, cells were suspended in diluted intracellular antibodies(PE-IL-17 (BD,USA), Alexa Fluor-IFN-γ (BD,USA), PE-isotype (BD,USA) or Alexa Fluor-isotype (BD,USA)), incubated 30 minutes at 4°C, then washed in 500uL 1X Perm Wash and resuspended in 100 uL of 1% paraformaldehyde. Data were collected using a BECKMAN COULTER Cytomics FC 500 Flow Cytometry System and analyzed with CXP software.

### Cytokine analyses in culture supernatants by ELISA

Cells were cultured and stimulated with the aforementioned antigens and controls for 24 hours (for IFN-γ responses) or 72 hours (for IL-17 responses) at 37°C in 5% CO_2_. Supernatants were collected and stored at -70°C until the time of assay. The concentration of IFN-γ was determined using the Human IFN-γ ELISA Set (eBiosciences, USA), after diluting the supernatants sufficiently to yield results within the linear range of the assay (routine dilutions were: PHA-stimulated samples, 1:5–1:100; PPD, 1:5–1:10) Supernatants from negative controls or ESAT-6 stimulated samples were not diluted. IL-17 concentrations in supernatants were quantified using the Human IL-17 ELISA Set (eBioscience, USA) with 72 hours of incubation. Supernatants from PHA-stimulated samples were diluted 1:2; all other assays were performed on undiluted supernatants. All assays were done in duplicate. Plates were read at 450 nm within 30 minutes of the end of the assay, using an ELISA plate reader.

### Statistical analyses

Differences between two groups were calculated by the unpaired, two tailed student’s t-test using Prism 5.01 software. The graph of severity grade of cavitary pulmonary tuberculosis was performed by statistical software SPSS 16.0. Values of p < 0.05 were considered statistically significant.

## Results

### Patient population

A total of 110 participants including 93 newly treated patients with PTB and 17 healthy control volunteers joined this study. All patients had their diagnoses confirmed by bacterial culture. 52 patients had cavitary lesions on chest CT scanning. Mean age in the cavitary group was 41.2±17.1 while that in the non-cavitary group was 39.9±17.4. No significant differences existed regarding age, sex, frequency of diabetes, history of BCG vaccination and other complications between the two groups.

Among 17 healthy volunteers, the mean age was 31.1±5.6. Five were excluded from healthy donors analysis due to their latent TB infection, given that their PBMC produced high levels of IFN-γ in response to ESAT-6 and positive TST skin test (Tuberculin Skin Test). We separated the data of five people as latent TB infection group. The data are shown in Table 1.

**Table 1:** Demographic Characteristics of 93 PTB patients.

	Groups (PTB)		
Characteristic	Cavitary	Non-cavitary	P value
	52(%)	41(%)	
sex (male)	35(67.3)	26(63.4)	0.69
age	41.17±17.11	39.93±17.37	0.23
extrapulmonary tuberuclosis	8(15.4)	8(19.5)	0.60
pleural tuberculosis	2	3	
bronchial tuberculosis	4	4	
intestinal tuberculosis	1	0	
laryngeal tuberculosis	1	0	
tuberculous meningitis	0	1	
Diabetes	9(17.3)	2(4.9)	0.07
Lesions ≥4 lung fields	22(42.3)	11(26.8)	0.12
Other complications (COPD, bronchiectasis)	3(5.8)	3(7.3)	0.76
history of BCG vaccination	48(92.3%)	36(87.8%)	0.47

### Similar levels, but large variation of IFN-γ responses in cavitary PTB and non-cavitary PTB

**Fig 1:**
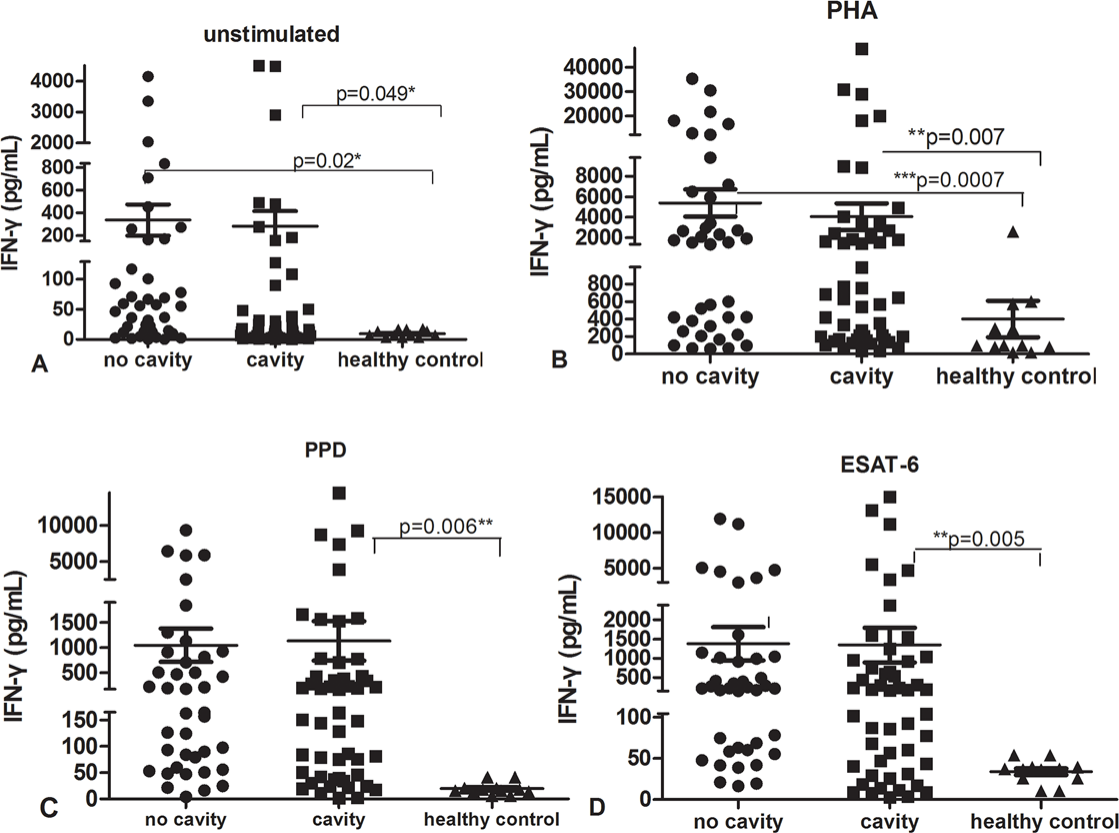
IFN-γ secretion by ELISA in supernatant of PBMC stimulated with medium (unstimulated, A), PHA(B), PPD(C) or ESAT-6(D). p value refers to comparison of IFN-**γ** levels.

Among 93 patients with active PTB including both the cavitary and non-cavitary groups, we observed large variation in IFN-**γ** responses stimulated by PHA, PPD, ESAT-6 or unstimulated control in both active PTB groups (Fig 1). As in our previous study, the degree of variation in IFN-**γ** responses to any of the stimuli in PBMCs from patients with active PTB was significantly higher than in those of healthy controls. For example, PPD stimulated IFN-**γ** secretion ranged from 14540 pg/mL to 2.31 pg/mL in cavitary PTB and from 9353 pg/mL to 3.89 pg/mL in non-cavitary PTB, and ESAT-6 stimulated IFN-**γ** varied over 700-5614-fold range, from 2.68 pg/mL to 15040 pg/mL in cavitary PTB and from 16.32 pg/mL to 11950 pg/mL in non-cavitary PTB. The variation of IFN-**γ** secretion was similar between cavitary and non-cavitary PTB.

The mean levels of IFN-**γ** in supernatants of PBMC stimulated with any of the stimuli, and in unstimulated cells, were also similar between the cavitary and non-cavitary PTB groups. However, the IFN-**γ** level in supernatant from healthy controls was significantly lower than that in either group with active PTB (p<0.05) except that in response to PPD and ESAT-6 from no cavity vs healthy control did not get statistic differences(p>0.05), it might result from limited number of healthy control group.

### Similar levels and variation of IL-17 secretion between cavitary and non-cavitary PTB

IL-17 secretion between cavitary and non-cavitary PTB groups was similar, p >0.05. In addition, we found that a wide range of IL-17 secretion existed in both active PTB groups but less variation as IFN-**γ** had. IL-17 ranged 1-350-fold in cavitary PTB and 1-110-fold in non-cavitary PTB.

**Fig 2:**
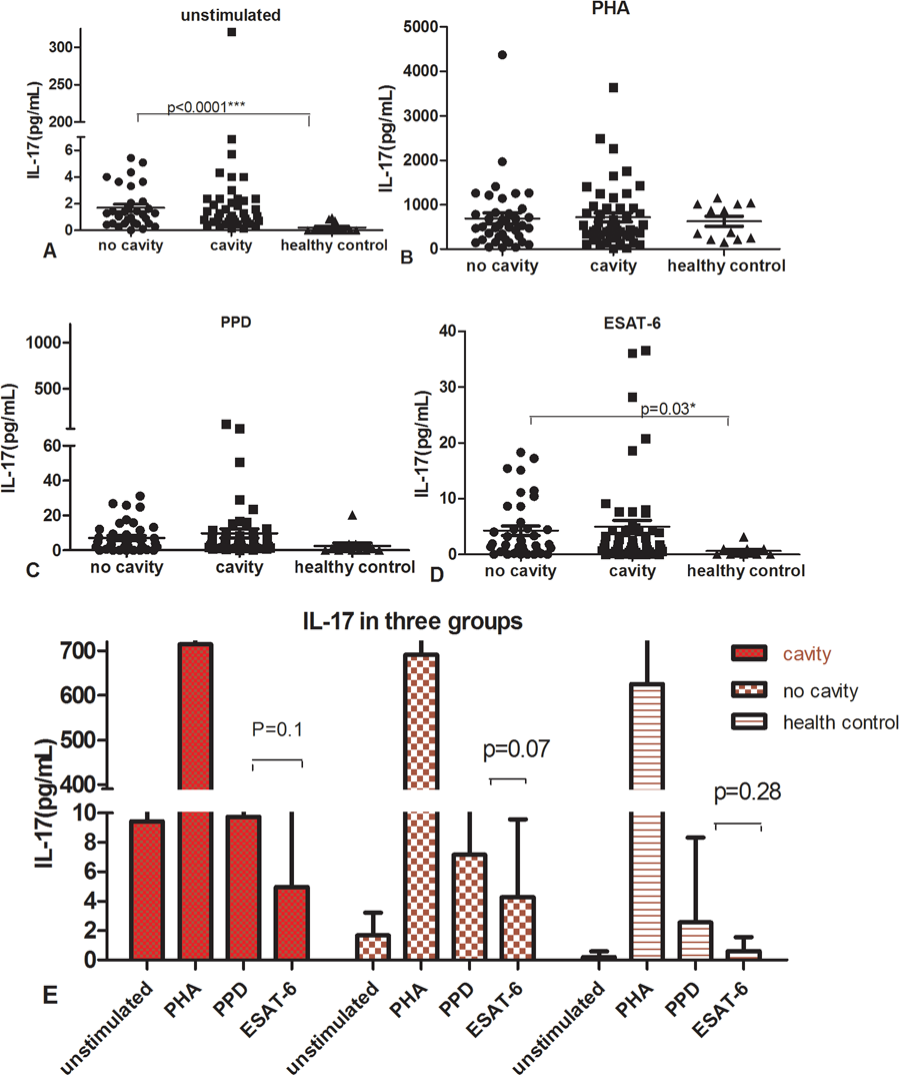
Shown in scatter diagram: IL-17 secretion tested by ELISA in supernatant of PBMC stimulated with medium (unstimulated, A), PHA (B), PPD(C) and ESAT-6(D). There were no statistic differences of IL-17 between cavitary and non-cavitary PTB. Fig 2E was showed in histogram: IL-17 secretion in response to different stimuli among patients with cavity, no cavity and healthy controls.

In supernatant stimulated by ESTA-6 and unstimulated, IL-17 secretions of non- cavitary PTB groups were significantly higher than those of healthy control group, p < 0.05. In PPD, ESAT-6 and PHA stimulation, IL-17 of cavitary PTB was slightly higher than that of non-cavitary PTB but there was no statistic significance as showed in Fig 2A–2D and Fig 2E showed the IL-17 change in different stimuli and different groups, there was no significant difference of IL-17 between PPD and ESAT-6 stimulation, p > 0.05.

### Similar percentages of Mtb antigen-specific IFN-γ and IL-17 producing CD4+ T cells between cavitary and non-cavitary PTB

**Fig 3:**
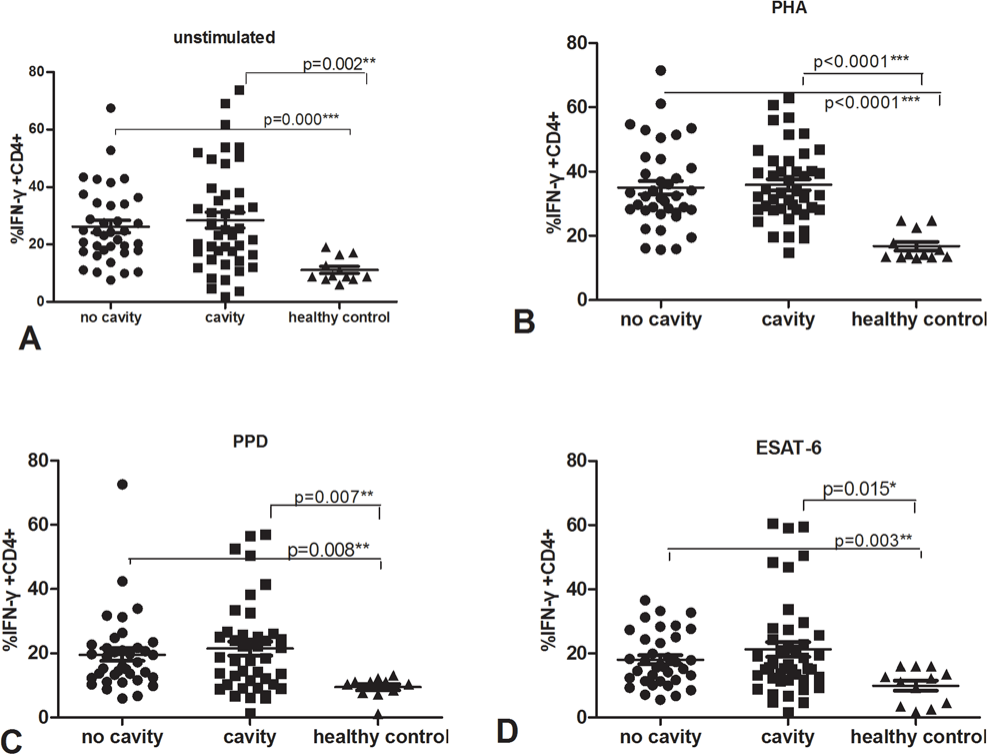
Percentage of IFN-γ secreting CD4+ T cells among cavitary and non-cavitary PTB as well as healthy controls, using cell staining and flow cytometry. PBMC cultured with unstimulated (A), PHA(B), PPD(C) and ESAT-6(D).

**Fig 4:**
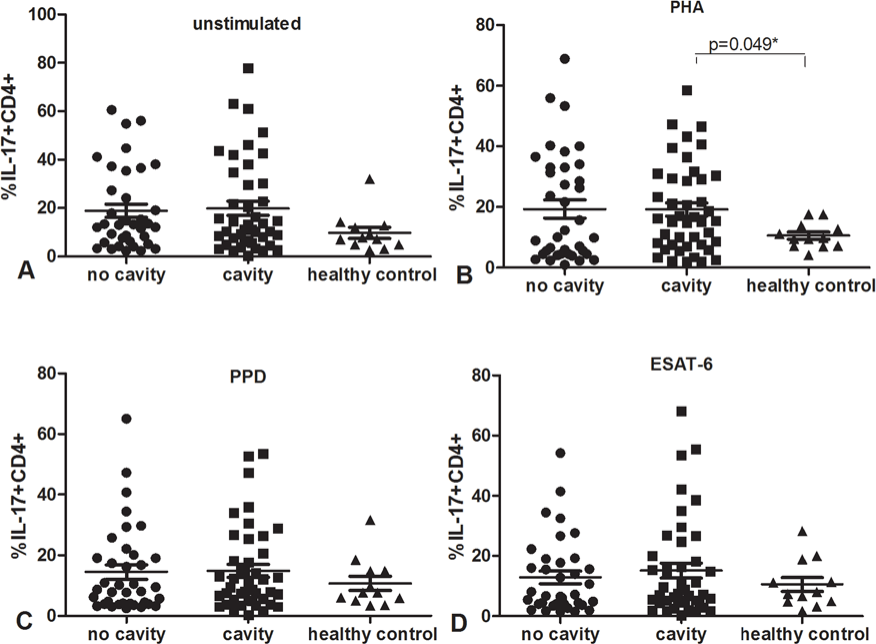
Percentage of IL-17 secreting CD4+ T cells among cavitary and non-cavitary PTB as well as healthy controls, using cell staining and flow cytometry. PMBC cultured with unstimulated (A), PHA(B), PPD(C) and ESAT-6(D).

We stained cells for identification by flow cytometry, then tested the percentage of IFN-**γ** and IL-17 producing CD4+ T cells after cell cultured and stimulated. The data was shown in Figs 3 and 4. The percentage of IFN-γ producing CD4+ CD3+ cells after stimulation with PHA, PPD, ESAT-6 and medium alone was significantly higher in both active PTB groups than in healthy donors, p<0.05. Furthermore, percentages of IFN-γ producing CD4+ CD3+ cells were similar in cavitary and non-cavitary PTB after any stimulus, percentages of IL-17 producing CD4+ T cells were also similar among cavitary PTB, non-cavitary PTB and healthy controls(p>0.05) except that of cavity TB under PHA stimulation was higher than that of healthy donors, p was 0.049.

### Mtb antigen-specific IFN-γ expression is decreased in PTB subjects with severe cavities

**Fig 5:**
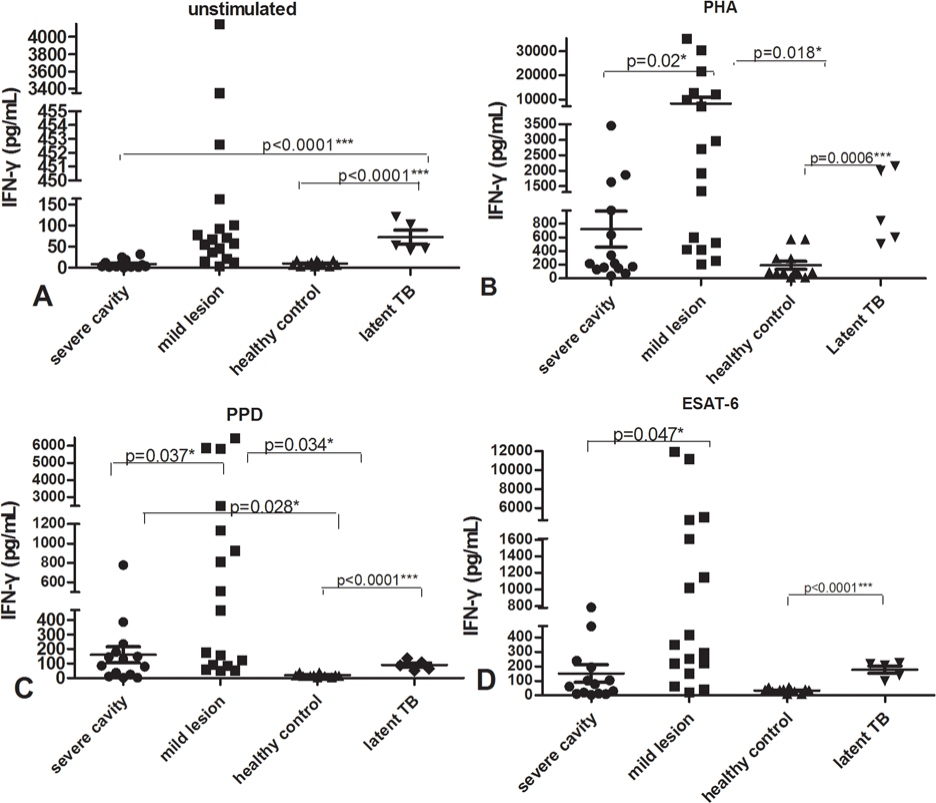
IFN-γ secretion measured by ELISA in supernatant of PBMC cultured with unstimulated (A), PHA(B), PPD(C), ESAT-6(D) in severe cavity PTB group, mild lesion non-cavity PTB group, latent TB infection group and control donors.

Since there were no significant differences in IFN-γ and IL-17 levels or IFN-γ and IL-17 producing CD4+ T cells between cavitary and non-cavitary PTB, we looked through all clinical characteristics of the subjects, then further subdivided the patients according to the diameter and number of cavities in cavitary PTB group and the field range of TB lesions on the chest CT in all patients. We discerned two subgroups (see S1–S2 Figs): the first subgroup (the severe cavity PTB group) included 14 cases, in which cavity diameter was > 3 cm or multiple (>3) cavities. The second subgroup (the mild non-cavity PTB group) included 17 cases, in which patients had mild lesions (≤ two lung fields) and had no cavity. The data (Fig 5A–5D) showed that the IFN-γ produced in response to PHA, PPD, and ESAT-6 in the severe cavity PTB group was statistically reduced compared with that in the mild lesion non-cavity PTB group (p values all < 0.05). IFN-γ stimulated by ESAT-6 and PHA in the severe cavity PTB group was as low as that in healthy controls and latent TB infection group(p>0.05), unstimulated IFN-γ level in severe cavity PTB was even significantly lower than that of latent TB group (p<0.0001), possibly indicating that the IFN-γ-producing T cells are impaired in the severe cavity group.

### Similar Mtb antigen-specific IL-17 and IFN-γ/IL-17 between severe cavitary PTB and mild non-cavity PTB

**Fig 6:**
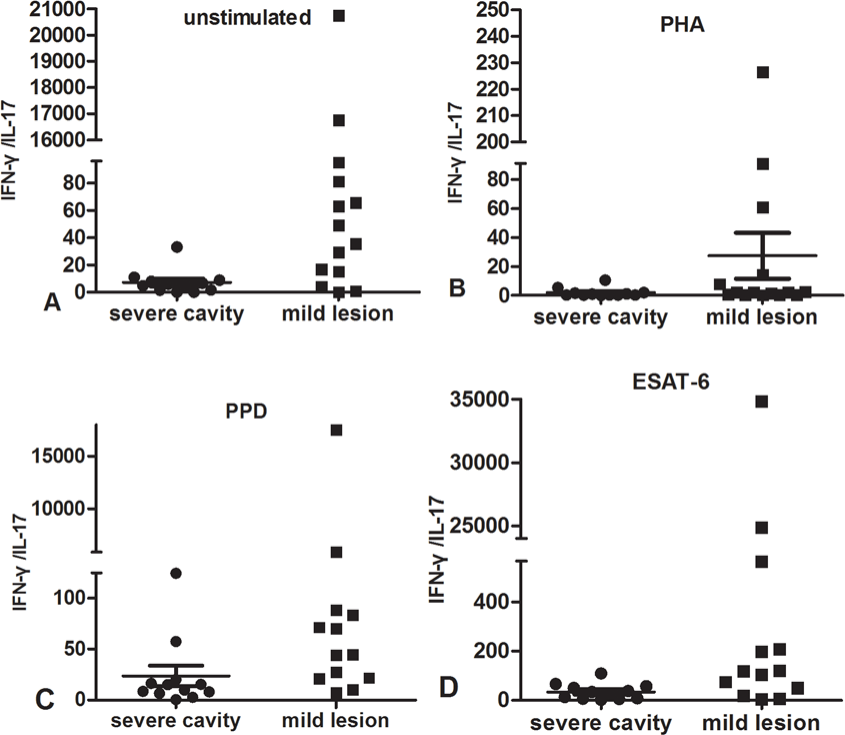
IFN-γ/IL-17 measured by ELISA in supernatant of PBMC cultured with unstimulated (A), PHA(B), PPD(C), ESAT-6(D) in severe cavity PTB group and mild lesion non-cavity PTB groups.

Since we found much lower IFN-γ responses in severe cavitary PTB subjects, we compared the differences of IL-17 responses to all stimuli between severe cavity and mild lesion non-cavity PTB. We measured similar levels of IL-17 in response to any stimuli between two groups (p>0.05). There was no significant different imbalance of IFN-γ/IL-17 although IFN-γ responses was impaired in severe cavity PTB, the data was showed in Fig 6.

### Mtb antigen-specific CD3+ percentages are remarkably decreased in severe cavitary PTB

**Fig 7:**
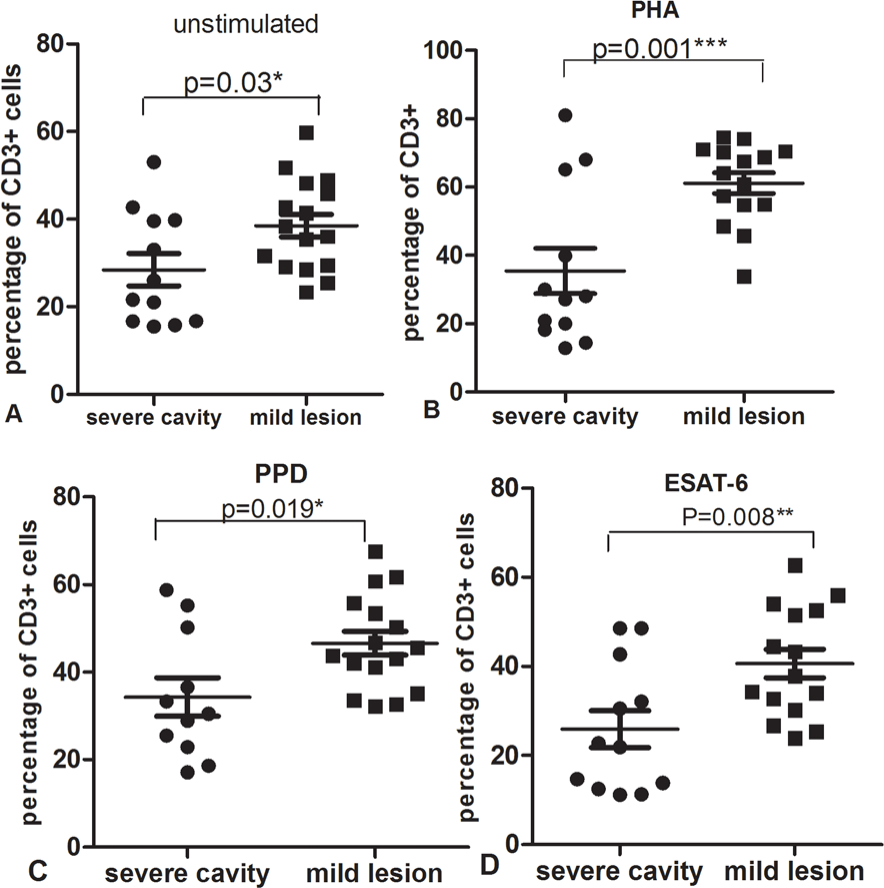
Percentage of CD3+ cells from PBMC after culture with unstimulated (A), PHA(B), PPD(C) or ESAT-6(D) in severe cavity PTB group and mild lesion non-cavity PTB group.

**Fig 8:**
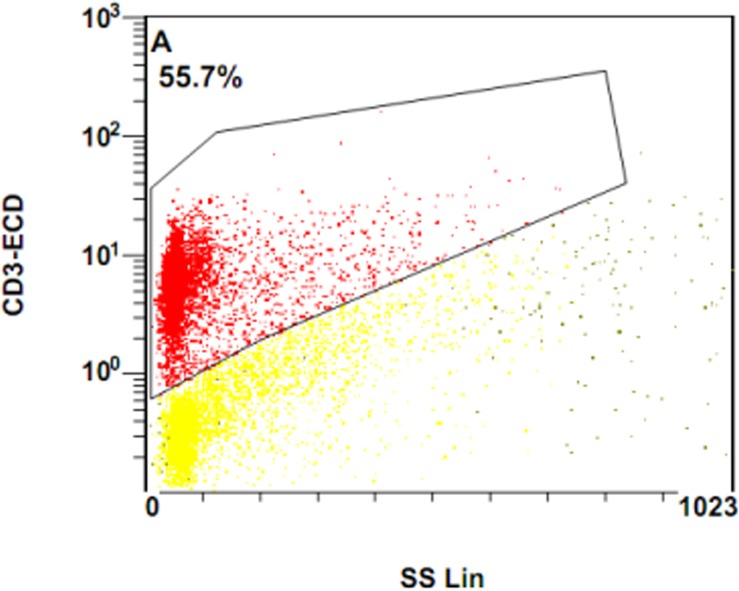
Plot image of CD3-ECD in flow cytometry in severe cavitary PTB.

**Fig 9:**
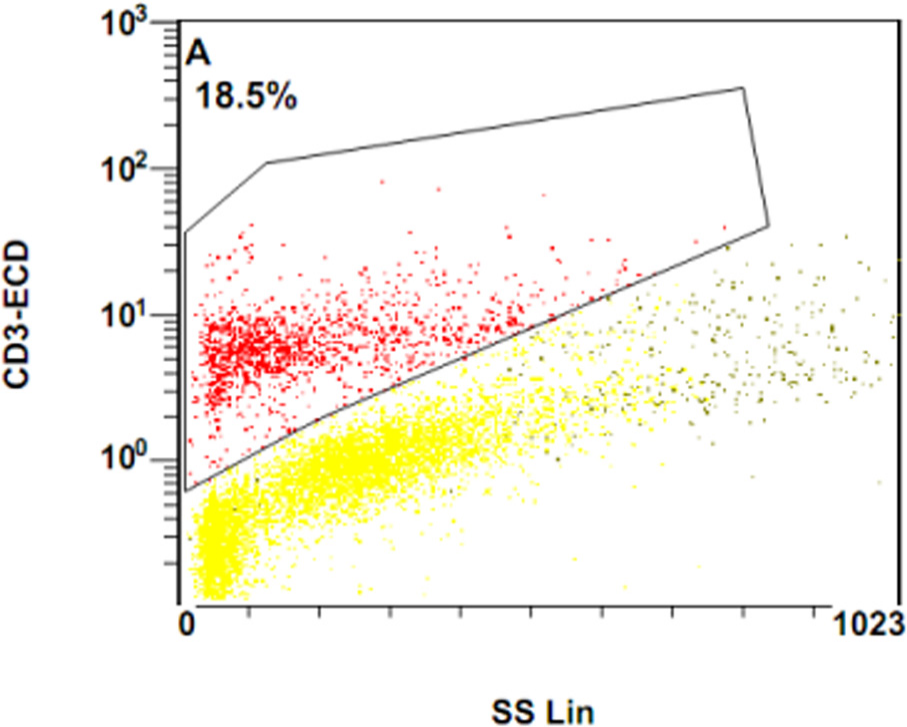
Plot image of CD3-ECD in flow cytometry in mild lesion non-cavity PTB(9).

**Fig 10:**
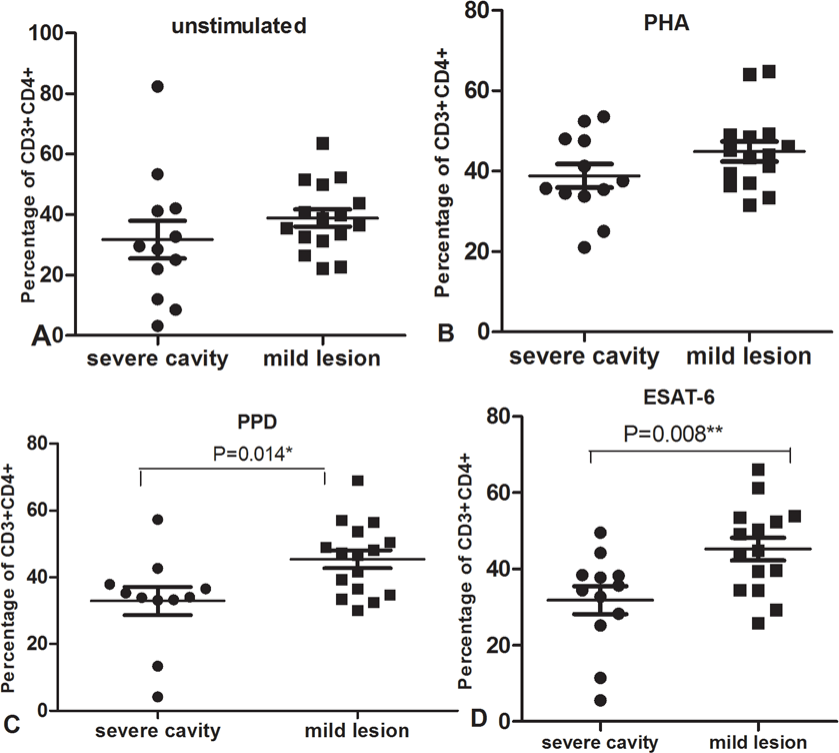
Percentage of CD3+CD4+ cells from PBMC after culture with unstimulated (A), PHA(B), PPD(C) or ESAT-6(D) in severe cavity PTB group and mild lesion non-cavity PTB group.

**Fig 11:**
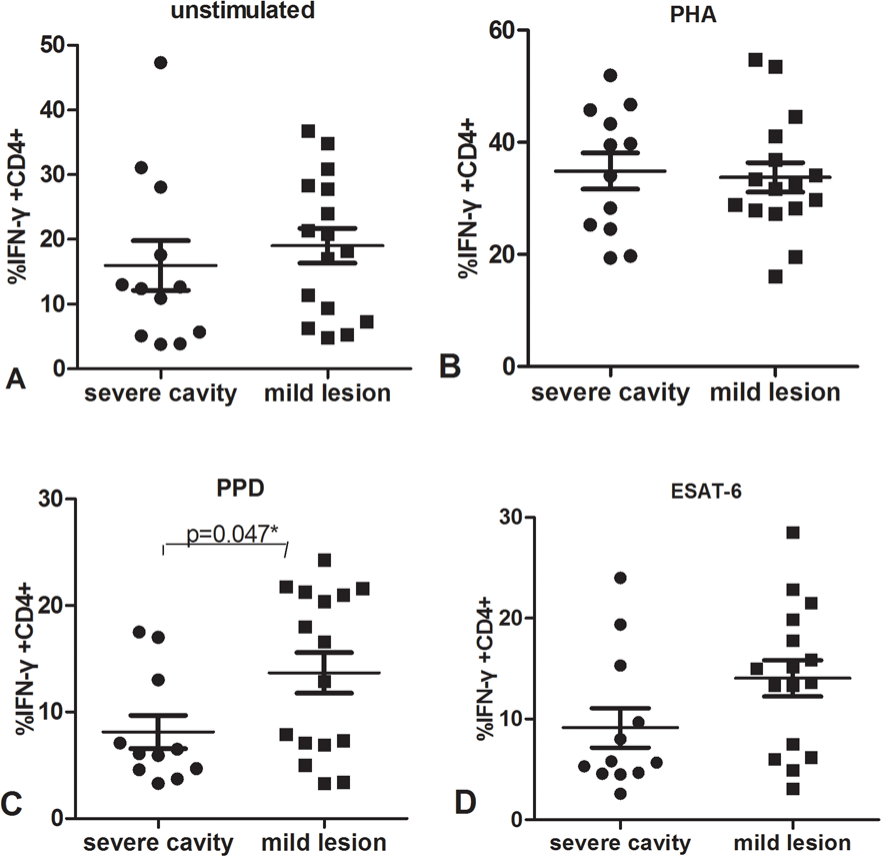
Percentage of IFN-γ+ CD4+ T cells from PBMC after culture with unstimulated (A), PHA(B),PPD(C) or ESAT-6(D) in severe cavity PTB groups and mild lesion non-cavity PTB group.

To further determine the potential reason for decreased IFN-γ in response to PHA and MTB antigens in severe cavity PTB, we compared the percentages of CD3+, CD3+CD4+, and CD3+CD8+ cells between two groups. The results were shown in Fig 7A–7D: the percentage of CD3+ cells in the severe cavity PTB group was much lower than in the mild lesion non-cavity PTB group, p values < 0.05. The plot images of percentages of CD3+ cells in two groups were shown in Figs 8 and 9. The decreased percentages of CD3+ cells in the severe cavity PTB group resulted in part from decreased CD3+CD4+ cells, rather than CD3+CD8+ T cells (p values < 0.05), data was shown in Fig 10A–10D. We also found significantly decreased PPD specific IFN-γ ^+^CD4^+^ expression in severe cavity PTB patients (p<0.05). IFN-γ ^+^CD4^+^ in response to ESAT-6 seemed like to be lower in severe cavity PTB but had no significant difference as shown in Fig 11, p>0.05.

### IFN-γ response changed and finally decreased with the severe cavity developed in lung of patient with PTB

**Fig 12:**
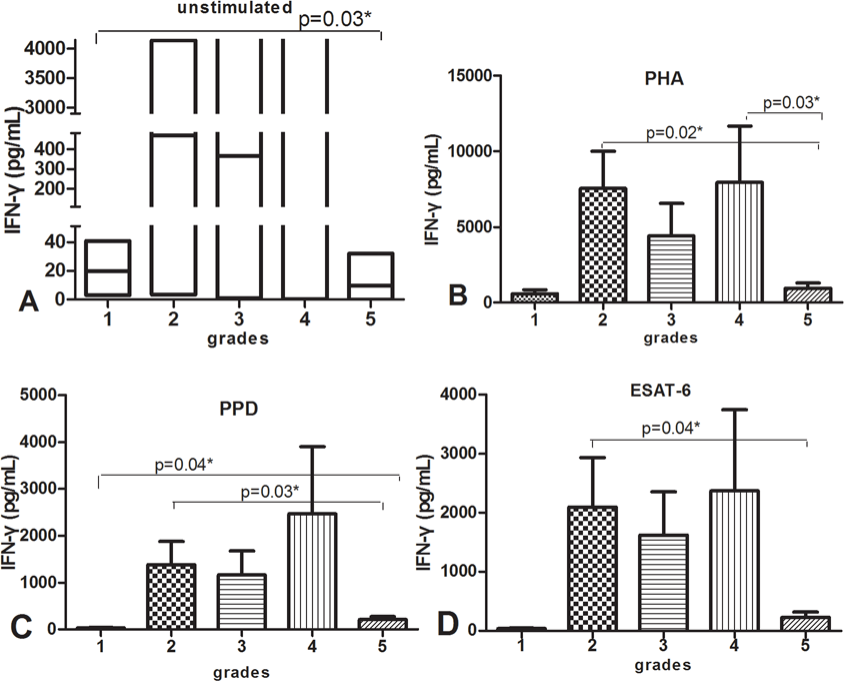
Shown in histogram, IFN-γ secretion by ELISA in supernatant of PBMC stimulated with medium (unstimulated, A), PHA(B), PPD(C) or ESAT-6(D) in five grades of subjects, p value refers to comparison of IFN-γ levels.

**Fig 13:**
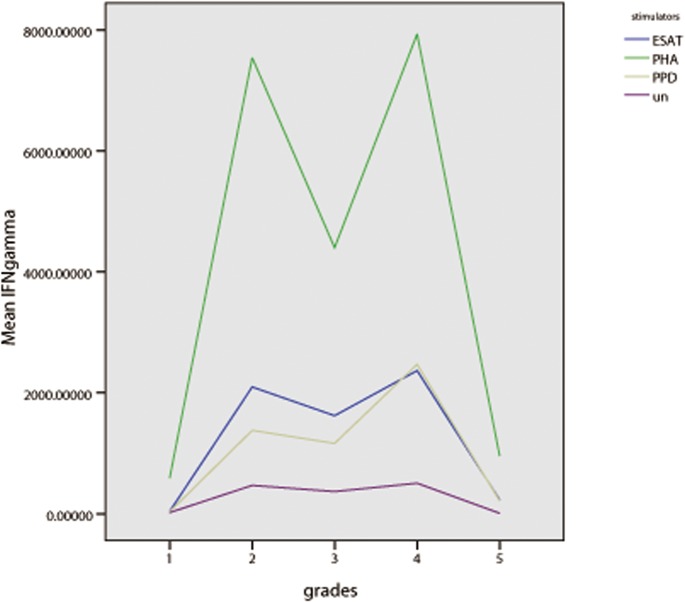
Shown in line graph, mean IFN-γ concentration in different groups of PBMC cultured with unstimulated, PHA, PPD and ESAT-6, horizontal axis represents different grades of patients, vertical axis represents mean IFN-γ secretion of PBMC. Grades represent cavitary severity of TB: 1 healthy controls; 2 no cavity and lesions≤2 lung fields; 3 cavity diameter≤2cm and lesions ≤ 3 lung fields; 4 2 cm < cavity diameter≤3cm or cavity diameter ≤2cm and lesions > 3 lung fields; 5 cavity diameter >3 cm or multiple cavities(cavity number ≥3).

We regrouped all the participants according to the severity of cavity and lesions in lungs, there were five grades for all patients, grade1: healthy control; grade 2: no cavity and lesions≤2 lung fields; grade 3: had cavity and cavity diameter≤2cm and lesions≤ 3 lung fields; grade 4: 2 cm < cavity diameter≤3cm or cavity number < 3 or cavity diameter ≤2cm but lesions > 3 lung fields; grade 5 cavity diameter >3 cm or multiple cavities(cavity number ≥3) with any severity of lung lesions. A total of 70 TB patients and 12 healthy donors were satisfied with above grading condition. We found that health controls with grade 1 had lower IFN-γ response to any stimuli, then IFN-γ response increased markedly 12–50 fold in grade 2 of patients with PTB, IFN-γ declined gradually about 20% in grade 3 and then slightly rose about 30% in grade 4, finally decreased 10–50 fold to be very low level in grade 5 which represented severe cavitary PTB, the change pattern of IFN-γ with severe cavities of TB was shown in Fig 12 in bar graph and Fig 13 in line graph.

## Discussion

This study reported the wide variation of *M*. *tuberculosis* antigen-specific IFN-γ and IL-17 responses in active PTB patients with the similarity in humans with latent TB infection shown in our previous study [21]. In the current study, *M*. *tuberculosis* antigen-specific IFN-γ and IL-17 in PBMC from patients with active PTB was significantly increased compared to that from cells of healthy controls or donors (HD). However, we did not find statistic differences of IFN-γ and IL-17 between cavity and non-cavity PTB groups.

For IL-17 expression in humans, we reviewed several published studies concerning IL-17 secretion in active TB patients. One study [23] reported that the proportion of IL-17 producing cells among lymphocytes was similar between TB patients and HD, but IL-17 producing cells in the gamma-delta T cell population were increased in PTB patients. Another study [24] found that *M*. *tuberculosis* induced IL-17 was markedly augmented in the blood of active PTB patients; moreover IL-17 expression in lymphocytes was higher in severe pulmonary lesions, reflecting the severity of lesions in the lungs. One recent study [25] showed that *ex vivo* PPD stimulation of PBMCs from patients with PTB did not significantly stimulate IL-17 release; rather, higher IL-17 was found in patients with infiltrative disease, in comparison with those affected with military and cavitary lesions. Our result implied some trend of increased IL-17 secretion in PBMC from cavitary PTB which was partly consistent with the aforementioned study, although we did not observe a statistical difference between cavity and non-cavity TB.

After staining the cells, we found similar levels of IFN-γ producing CD4+CD3+ cells between cavity and non-cavity PTB; both groups had statistically higher percentages of IFN-γ producing CD4 T cells than HD. Through comparison of IL-17 levels in PBMC by ELISA and producing T cells by flow cytometry, we came to the conclusion that TB antigen-specific IL-17 secretion was not statistically increased in PBMC of active PTB.

Since we did not find any difference of levels in IFN-γ and IL-17 secretion between cavity and non-cavity PTB, we looked through the detailed data from each patient because so much individual variation existed. Using chest CT imaging, we defined severe cavitary PTB by the presence of multiple cavities (≥ 3) or a cavity diameter of more than 3 cm, while mild non-cavitary PTB was defined as non-cavity TB with less than two lung fields of lesions. We found that IFN-γ in response to PHA and TB antigens in severe cavitary PTB patients was statistically lower than that in patients with mild lesions of non-cavity PTB. When we further analyzed the staining data, significantly decreased CD3+ percentage in severe cavitary PTB compared to that in mild lesion non-cavity PTB also was observed. In addition, the decrease in CD3+ T cells resulted from a reduction in CD3+CD4+ cells, rather than in CD3+CD8+ cells.

To further elucidate and expose the relationship of IFN-γ response and the severity of cavity of TB in lungs, we graded the enrolled participants as grade 1–5 according to the severity of cavity and pulmonary lesions. IFN-γ in response to any stimuli increased dramatically in grade 2 represented PTB subjects compared to the level of grade 1 which represented heathy control, then slowly went down in grade 3 and slowly went up in grade 4, finally decreased to the very low level which was almost as low as that in healthy control shown in Fig 10, this figure of change trend further demonstrated that IFN-γ depression in PBMC of PTB was associated with cavitary severity of TB, the decreased and very low IFN-γ response will be observed when PTB develops to severe cavities.

Barry, et al [11] demonstrated impaired antigen-specific CD4+ T lymphocytes in cavitary tuberculosis, and other studies [26,27] also reported impaired antigen specific IFN-γ in cavitary tuberculosis, but no one made the stratification analysis for cavitary PTB by number and diameter of cavities, their numbers of participants enrolled were much lower than current study. At the site of infection, the proliferation reaction and immune response were also weaker in cavitary tuberculosis as reported by two studies [28, 29].

To explain our study results, we postulate several mechanisms to explain the decreased CD3+ percentage in PBMC of severe cavitary PTB after restimulatioin with *M*. *tb* antigens *in vitro*. Firstly, CD3+ T cells might manifest weak viability as a decreased percentage of CD3+ cells, as observed in cultivation with PHA, which is a non-specific polyclonal stimulant of T cells. Excessive apoptosis might occur in CD3+ T cells during the course of contact between antigens and cells [30], as recent studies showed increased apoptosis in active PTB. Using PD-1 and its ligands as indicators of measurement, a higher percentage of apoptotic T cells and CD4 lymphocytes in children with acute phase central nervous tuberculosis has been observed [31,32]. Secondly, the decreased percentage of CD3+ T cells might have been due to excess accumulation of neutrophils in severe cavitary PTB[11,33], because previous studies [34] had pointed out that accumulation of neutrophils was detrimental to the host and associated with loss of IFN-γ responsiveness. We did not measure neutrophil numbers in this study so this possibility needs to be verified in future research. Thirdly, we are not able to tell whether there are altered immune responses at the site of TB infection because we failed to separate large numbers of cells from Bronchoalveolar lavage fluid (BALF) of active PTB. BALF might contain excess IL-17 and neutrophils in severely cavitary PTB, which may have different proportions of cells compared with mild lesion non-cavity PTB. That was a weakness of this study. We need to further measure and analyse additional immune responses (Th1, Th2, Th17, Treg) and interactions between lymphocytes, neutrophils and DC cells. Treg populations would be possibly involved with the loss of Ag-specific IFN-γ in severe cavities PTB and might also support the immune disruption that occurs.

In summary, the wide range of IFN-γ in active PTB patients was observed. IFN-γ and IL-17 secretion were similar ex vivo between cavitary PTB and non-cavitary PTB, and IL-17 seemed to have no association with cavities in blood of active PTB. The IFN-γ response increased greatly at the mild stage of PTB, then changed with the progress of the lesions and finally dropped to very low level when lesions make progress to be at stage with severe cavities. We demonstrate that M.tuberuclosis antigen-specific Th1 response is impaired when PTB lesions develop to severe cavities.

## Supporting Information

S1 Fig Chest CT image in severe cavitary PTB, large cavity 7.5cm in diameter in left upper lobe marked by a white arrow. (TIF)

S2 Fig Chest CT image in mild lesion non-cavitary PTB, mild lesion without cavity confined to posterior segment of left upper lobe marked by a white arrow. (TIF)
