# Supplementary figures and images for: Impaired M. tuberculosis Antigen-Specific IFN-γ Response without IL-17 Enhancement in Patients with Severe Cavitary Pulmonary Tuberculosis
Source: PLoS One. 2015 May 27;10(5):e0127087. doi: 10.1371/journal.pone.0127087 (PMC4446217; doi:10.1371/journal.pone.0127087)

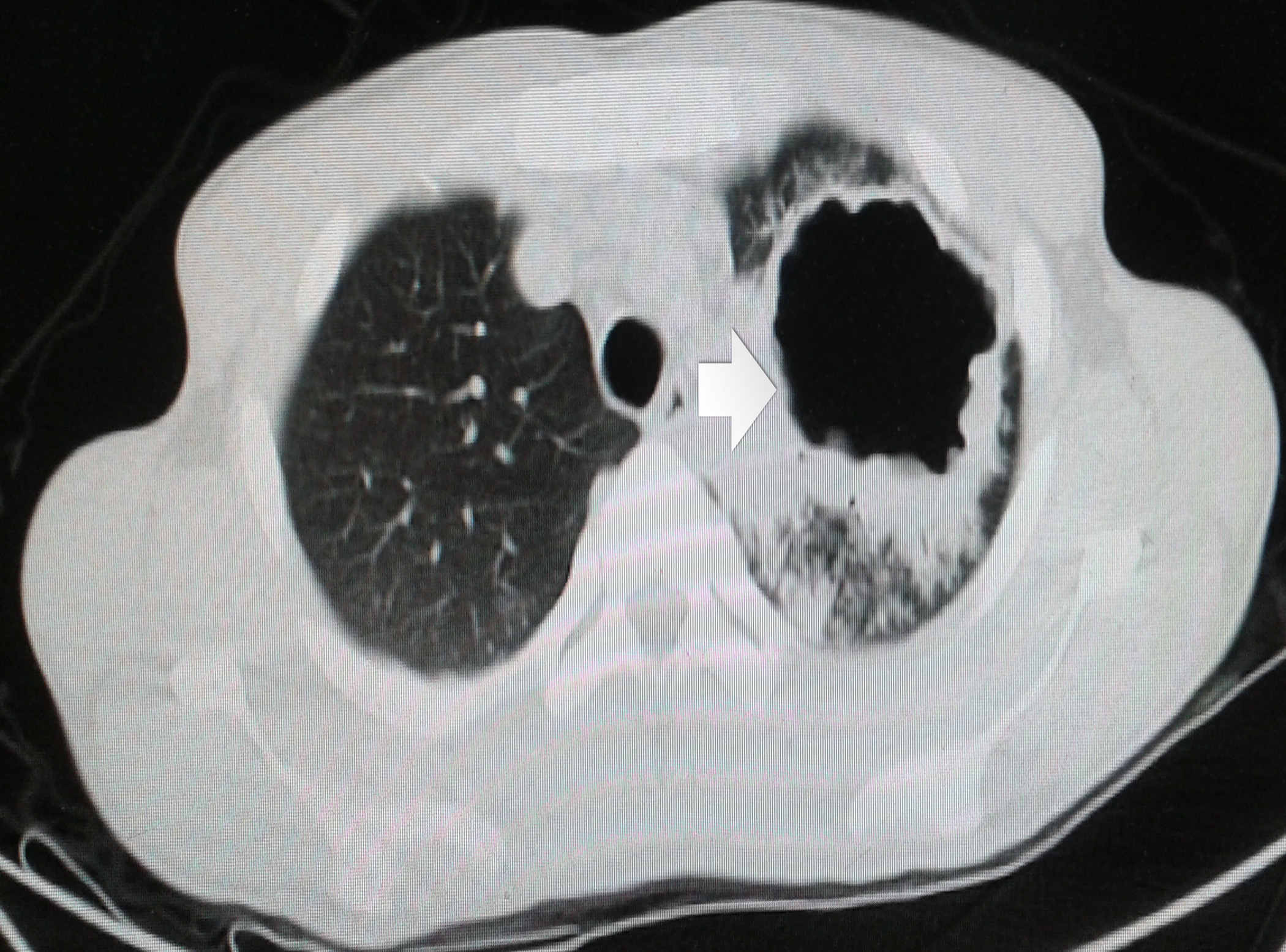

Supplement: S1 Fig — (TIF) [file pone.0127087.s001.tif]

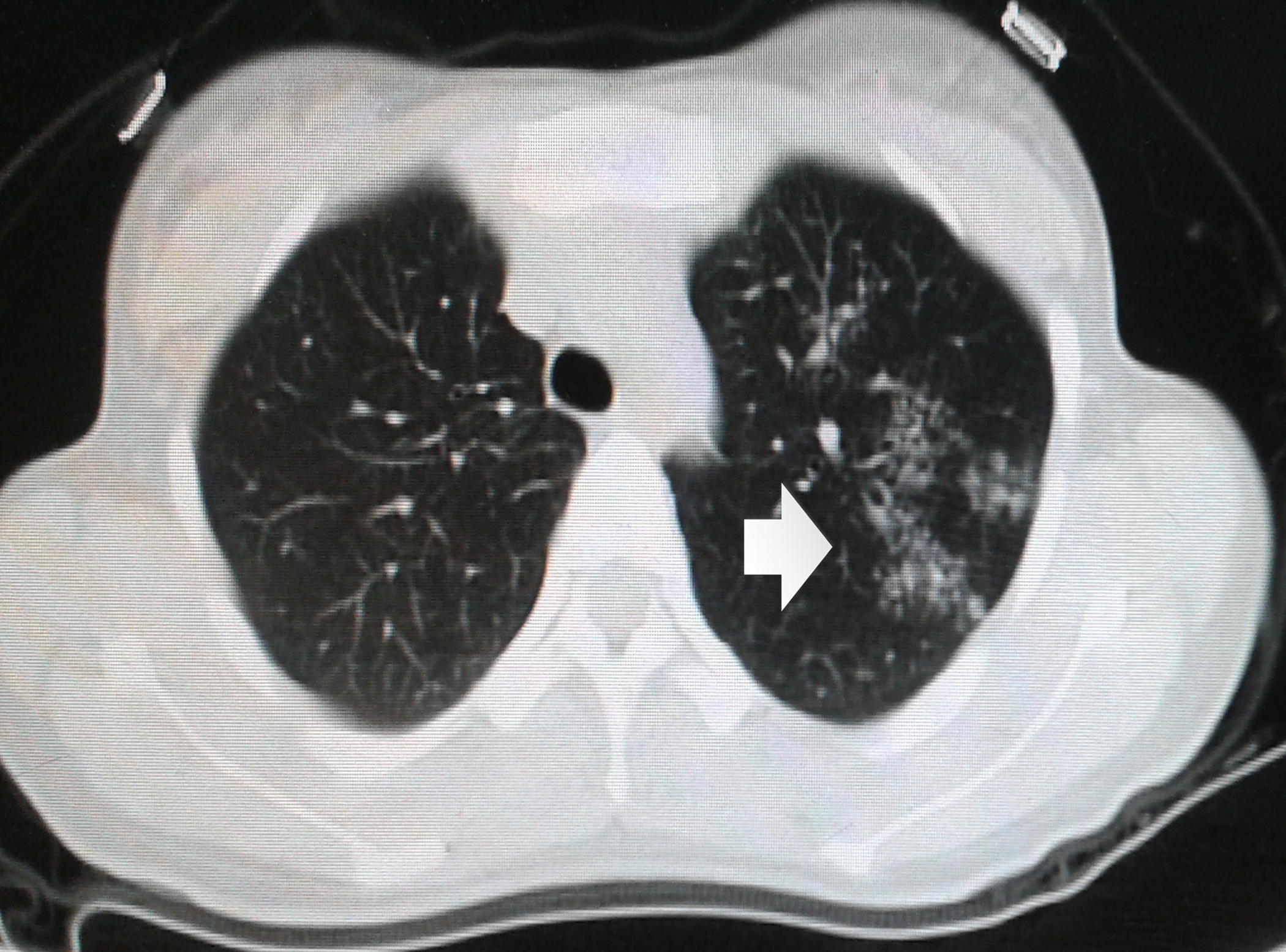

Supplement: S2 Fig — (TIF) [file pone.0127087.s002.tif]
